# Expansion of Granulocytic, Myeloid-Derived Suppressor Cells in Response to Ethanol-Induced Acute Liver Damage

**DOI:** 10.3389/fimmu.2018.01524

**Published:** 2018-07-19

**Authors:** Sha Li, Ning Wang, Hor-Yue Tan, Ming Hong, Man-Fung Yuen, Huabin Li, Yibin Feng

**Affiliations:** ^1^Li Ka Shing Faculty of Medicine, School of Chinese Medicine, The University of Hong Kong, Pokfulam, Hong Kong; ^2^Division of Gastroenterology and Hepatology, Queen Mary Hospital, Department of Medicine, Li Ka Shing Faculty of Medicine, The University of Hong Kong, Pokfulam, Hong Kong; ^3^Guangdong Provincial Key Laboratory of Food, Nutrition and Health, School of Public Health, Sun Yat-sen University, Guangzhou, China

**Keywords:** granulocytic myeloid-derived suppressor cells, acute alcoholic liver damage, YAP, IL-6, immune suppression

## Abstract

The dual role of ethanol in regulating both pro-inflammatory and anti-inflammatory response has recently been reported. Myeloid-derived suppressor cells (MDSCs) are one of the major components in the immune suppressive network in both innate and adaptive immune responses. In this study, we aim to define the role of a population expressing CD11b^+^Ly6G^high^Ly6C^int^ with immunosuppressive function in response to ethanol-induced acute liver damage. We find this increased granulocytic-MDSCs (G-MDSCs) population in the blood, spleen, and liver of mice treated with ethanol. Depletion of these cells increases serum alanine aminotransferase and aspartate aminotransferase levels, while G-MDSCs population adoptive transfer can ameliorate liver damage induced by ethanol, indicating the protective role in the early stage of alcoholic liver disease. The significant changes of T-cell profiles after G-MDSCs populations adoptive transfer and anti-Gr1 injection signify that both cytotoxic T and T helper cells might be the targeted cells of G-MDSCs. In the *in vitro* study, we find that myeloid precursors preferentially generate G-MDSCs and improve their suppressive capacity *via* chemokine interaction and YAP signaling when exposed to ethanol. Furthermore, IL-6 serves as an important indirect factor in mediating the expansion of G-MDSCs populations after acute ethanol exposure. Collectively, we show that expansion of G-MDSCs in response to ethanol consumption plays a protective role in acute alcoholic liver damage. Our study provides novel evidence of the immune response to acute ethanol consumption.

## Introduction

The detrimental effects of ethanol on health are numerous, and its most important side effect is damage to the liver (1, 2). Ethanol consumption initiates immune response that may lead to liver damage and consequently, alcoholic liver disease (ALD) (3, 4). In the course of ALD, the infiltration of immune cells like neutrophils, macrophages, T cells, and B cells further induces pro-inflammatory cytokines and chemokine, resulting in hepatic inflammation and tissue damage (5–8). However, recent evidence indicates that ethanol may comprehensively regulate immune response (1, 9, 10). Ethanol exposure might mediate immune responses along a spectrum that spans from pro-inflammatory to anti-inflammatory and from damage to resolution *via* unidentified mechanisms. Acute ethanol consumption drives the initial pro-inflammatory immune response. Afterward, anti-inflammatory response would be promoted to protect the host from the systemic cytokine storm (11, 12). Cellular self-protective mechanisms against ethanol-induced detrimental effects have been proposed, but have not yet been proven and elaborated on.

Identified as a heterogeneous population of immature myeloid cells, myeloid-derived suppressor cells (MDSCs) are one of the major components in the immune suppressive network to both innate and adaptive immune response (13, 14). They have been divided into granulocytic-MDSCs (G-MDSCs) and monocytic-MDSCs (M-MDSCs) in rodents based on the differential expression of Ly6G or Ly6C (15). G-MDSCs and M-MDSCs with different morphology have immune suppressive abilities *via* different pathways (16). The immunosuppressive capacity of MDSCs is generally attributed to upregulated expression of immune suppressive factors such as arginase-1 and iNOS, as well as an increase in nitric oxide and ROS in immature status (17, 18). A variety of factors have been reported to be involved in the expansion and activation of MDSCs (19–21). Of note, the Janus kinase/signal transducer and activator of transcription (JAK/STAT) pathway activated by factors such as IL-6 has a vital role in mediating both the expansion of MDSCs and their immune suppressive function (22). STAT3 mediates the expansion and accumulation of MDSCs primarily by stimulating myelopoiesis and inhibiting differentiation of immature myeloid cells *via* upregulation of S100A8/9, and it fosters survival of MDSCs by inducing the expression of myc, B-cell lymphoma XL (BCL-XL), and cyclin D1 (22–24). There have been several advances in understanding the molecular mechanisms governing MDSCs accumulation as well as identification of their detrimental role in facilitating the escape of tumor cells from immune surveillance (18); however, it is only in recent years that their protective function has been highlighted in several pathological conditions (25–29). Notably, in the context of acute hepatitis, MDSCs can limit immunogenic T-cell responses and subsequent tissue damage (30). A study showed that chronic ethanol consumption enhances MDSCs in B16BL6 melanoma-bearing mice (31). However, the role of MDSCs in ethanol-induced liver damage remains unclear.

In the present study, we tried to identify the profile of MDSCs in response to acute ethanol consumption. Currently, the definition of CD11b^+^Ly6G^+^ population is still controversial. Both neutrophils and G-MDSCs express CD11b and Ly6G (32). The phenotypic, morphological, and functional heterogeneity of these cells generates confusion in the investigation and analysis of their roles in inflammatory responses (33). Cells expressing CD11b^+^Ly6G^+^ with T-cell immune suppressive activity usually would be considered as G-MDSCs, which includes some neutrophils having immune inhibitory functions (33, 34). It has also been proposed that G-MDSCs might represent novel phenotypes of neutrophils with immune suppression. We hypothesized that this G-MDSCs played a hepatoprotective role in alcoholic injury. To test this hypothesis, loss- and gain-of-function analyses of G-MDSCs after acute ethanol exposure were performed. The cytoprotective role of G-MDSCs in acute alcoholic liver injury has been illustrated. Direct and indirect factors that mediate expansion of MDSCs upon acute ethanol consumption have been identified. As IL-6/STAT3 signaling has been intensively implicated in inducing MDSCs, particular attention was paid to this signaling pathway and its down-stream target S100A8.

## Materials and Methods

### Mice and Tissue

Six- to eight-week-old male mice (C57BL/6) were administered by gavage a single dose of ethanol (6 g/kg body weight). The ethanol solution used is a mixture of pure ethanol with ddH_2_O and the final percentage is 50% (vol/vol). The gavage volume (μL) of 50% (vol/vol) ethanol solution for each mouse = mouse body weight in grams × 15. Control mice were given isocaloric maltose dextrin solution. The gavage volume (μL) of 72.0% (wt/vol) maltose dextrin solution for each mouse = mouse body weight in grams × 15. Mice were sacrificed after ethanol administration. Blood, liver, spleen, and bone marrow were collected for further analysis. All experimental protocols involving mice were approved by the Committee on the Use of Live Animals in Teaching and Research of The University of Hong Kong, Hong Kong.

### Preparation of Single-Cell Suspension

Cells were prepared as previously described (29, 35).

(1) Blood samples were withdrawn by cardiac puncture when the animals had been anesthetized with ketamine/xylazine mixture (ketamine 100 mg/kg, xylazine 10 mg/kg, i.p.), then quickly mixed with heparin (20 IU heparin per mL blood). Samples were stained with appropriate antibodies for 15 min at room temperature. Following incubation, 1 mL 1× RBC lysing buffer was added for 5 min at room temperature. Thereafter, centrifugation at 300 *g* for 5 min at 4°C and wash the pellet by PBS. Finally, the cells were re-suspended in 0.3 mL PBS supplemented with 1% FBS for flow cytometer analysis.
(2) Liver tissue was minced into small pieces with surgical scissors. Then it was forced gently through a 200 µm-gauge stainless steel mesh *via* a sterile syringe plunger and suspended in 5 mL RPMI-1640 medium. The suspension was centrifuged at 528 *g* for 10 min at 4°C. The obtained pellet was re-suspended in 5 mL type IV collagenase solution (1 mg/mL dissolved in RPMI-1640 medium) and thereafter incubated at 37°C with shaking (100 rpm) for 30 min at 37°C. After incubation, 3 mL RPMI-1640 medium was added to the digested suspension and kept on ice for 5 min, and thereafter collected the top 3 mL suspension and centrifuged at 528 *g* for 10 min at 4°C. The resulting pellet was re-suspended in 10 mL 36% Percoll in Hank’s buffered salt solution and then centrifuged at 850 *g* with the off-brake setting for 30 min at 25°C. After centrifugation, the pellets containing various non-parenchymal cells thus obtained were re-suspended in 2 mL 1× RBC lysing buffer, incubated for 5 min at room temperature, then centrifuged at 480 *g* for 8 min at 8°C. Finally, the pellet obtained was re-suspended in PBS and subjected to antibodies staining for flow cytometer analysis.
(3) Splenic tissue was minced in RPMI-1640 medium and transferred to pass through 70 µm cell strainer. The harvested single-cell suspension was pelleted by centrifugation at 300 *g* for 5 min. The red blood cells were eliminated by incubating in 1× RBC lysis buffer for 5 min at room temperature. After lysis and centrifugation, cell pellets were re-suspended in PBS and subjected to antibodies staining.
(4) Bone marrow cells were collected from femurs and tibias of the mice. Bone marrow cells were flushed from both ends of the bone shafts by using a 25-G needle and a 3-mL syringe filled with RPMI-1640 supplemented with 10% FBS. After passing through 70 µm cell strainer, bone marrow cell suspension was obtained. Overlaid the bone marrow cell suspension slowly over Lymphoprep™ density gradient medium and centrifuged for 30 min at 2,300 rpm with off-brake setting at 4°C. The interface layer between RPMI-1640 medium and density gradient medium was collected and pelleted by centrifugation at 300 *g* for 5 min at 4°C. Bone marrow-derived cells including mononuclear cells and polymorphonuclear leukocytes re-suspended in PBS and subjected to antibodies staining.

### Flow Cytometry

Cells were uniformly divided into different tubes and stained with specific anti-mouse antibodies for 15 min at room temperature in the dark. In tubes for determination of the population of MDSCs, APC-conjugated anti-CD11b antibody, FITC-conjugated anti-Ly6G antibody (clone 1A8, eBioscience), and PE-Cy7-conjugated anti-Ly6C antibody were co-stained. In tubes for the determination of population of T cells, APC-conjugated anti-CD3 antibody, FITC-conjugated anti-CD4 antibody, and APC-Cy7-conjugated anti-CD8 antibody were co-stained. In tubes for the determination of proliferation of T cells, Pacific Blue-conjugated anti-CD3 antibody, PE-conjugated anti-CD4 antibody, and APC-Cy7-conjugated anti-CD8 antibody were co-stained. In tubes for bone marrow progenitors, PE-conjugated anti-CD115 antibody, APC-conjugated anti-CD64 antibody, and FITC-conjugated anti-CD34 antibody were co-stained. The detailed conjugated antibody panels were listed in Table S1 in Supplementary Material. Corresponding isotype antibodies were applied as controls. After incubation, cells were pelleted by centrifugation at 300 *g* for 5 min at 4°C. Then pellets were washed by PBS for twice. Finally, all cells were re-suspended in PBS supplemented with 1% FBS and subjected to flow cytometer analysis. The analysis was performed on FACS Canto II cytometer (BD) or LSR Fortessa (BD), and cells were sorted by FACS Aria I cytometer (BD). For each analysis, about 2,000 events of targeted population were gated. FlowJo software was used to analyze the data. For all sorted cells, cells purity was further determined by flow cytometer and the results showed that the purity of sorted cells was over 90%.

### Measurement of Cytokines

Granulocytic-MDSCs cells were sorted from the livers of mice receiving vehicle or alcohol and then cultured in RPMI-1640 for 48 h. LEGENDplex™ Mouse Inflammation Panel (13-plex) was used for the measurement of IL-10 and GM-CSF in the supernatants (San Diego, CA, USA). The determination was processed according to the kit procedure. BD™ Cytometric Bead Array mouse IL-6 and TNF-α Flex Set were used for the serum measurement of IL-6 and TNF-α.

### Depletion of G-MDSCs *In Vivo*

Anti-Gr1 (RB6-8C5) or isotype IgG2b antibody were injected i.p. into mice at a dose of 120 mg per mouse. About 12 h later, mice were treated with ethanol at a dose of 6 g/kg.

### G-MDSCs Adoptive Transfer Study

CD11b^+^Ly6G^high^Ly6C^int^ cells were purified from the bone marrow of naïve mice and about 1 × 10^7^ cells in 200 µL PBS were injected into recipient mice *via* i.p. The control group mice received an i.p. injection of 200 µL PBS. Then, these mice were treated with ethanol at a dose of 6 g/kg and sacrificed. The viability of transferred cells was >95%, as determined by trypan blue exclusion assay.

### T Cells Adoptive Transfer of T Cells

T cells including CD3^+^CD4^+^ and CD3^+^CD8^+^ were purified from the spleen of naïve mice by FACS Aria I cytometer (BD). About 1 × 10^7^ cells (7 × 10^6^ CD3^+^CD4^+^ cells and 3 × 10^6^ CD3^+^CD8^+^ cells) in 200 µL PBS were injected into recipient mice *via* tail vein injection. The control group mice received 200 µL PBS *via* tail vein injection. Then, these mice were treated with ethanol at a dose of 6 g/kg and sacrificed. The viability of transferred cells was >95%, as determined by trypan blue exclusion assay.

### *In Vitro* Culture of Bone Marrow-Derived Cells

Bone marrow-derived cells were collected from femurs and tibias of mice by above-mentioned protocol and cultured in RPMI 1640. Ethanol was added at concentration of 0.5%, 2.5% (vol/vol) to study its impact on G-MDSCs. In experiments for IL-6 *in vitro*, IL-6 antibody (ThermoFisher, 40 ng/mL) or isotype antibody was added into the medium accordingly. In experiments for YAP inhibition *in vitro*, YAP inhibitor verteporfin (Tocris, 2 µg/mL) or vehicle was added into the medium accordingly.

### Carboxyfluorescein Succinimidyl Ester (CFSE) Labeling

CD8^+^ T cells were sorted from spleens using FACS Aria I cytometer (BD). After two washes with PBS, purified CD8^+^ T cells were adjusted to 2 × 10^6^ cells/mL and mixed quickly with an equal volume of 10 µM CFSE solution. After a 10-min incubation in the dark, 10 volumes of cold PBS were added to terminate the reaction. Then, cells were washed twice with PBS and adjusted to 1 × 10^6^ cells/mL.

### T-Cell Suppression Assays

In a cell culture plate coated with 5 µg/mL anti-CD3 antibody, 2 × 10^5^ CD8^+^ T cells with CFSE labeling were cultured in RPMI-1640 supplemented with 5 µg/mL anti-CD28 antibody and 10% FBS for 96 h. In some wells, CD8^+^ T cells were co-cultured with 2 × 10^5^ purified CD11b^+^Ly6G^high^Ly6C^int^ cells or CD11b^+^Ly6G^low^Ly6C^high^ from the liver of mice treated with ethanol or vehicle, or mice receiving G-MDSCs populations adoptive transfer. In separated experiments about IL-10R blockade, CD210 antibody (BD Pharmingen™, 1 µg/mL) or isotype control (rat IgG1) was added in to the culture medium. The cells were harvested following culturing and stained with Pacific Blue-conjugated anti-CD3 antibody. As the CFSE signal was diluted with each cell division, cells exhibiting low fluorescence intensity of CFSE were considered to have proliferated. Thus, % suppression was calculated as 100% − (cells with low CFSE fluorescence intensity) %.

### BrdU Staining With Anti-BrdU Antibody

Bone marrow-derived cells were cultured with ethanol (vol/vol: 0.5%) *in vitro* for 5 h. Afterward, cells were first labeled with BrdU at a final concentration of 10 µM and incubated for 30 min in a CO_2_ incubator at 37°C. After cells were washed and centrifuged, 100 µL of the cell suspension was incubated with mouse Anti-BrdU Alexo Fluor 488 antibody, anti-CD11b APC, and anti-Ly6C PE-Cy7 at the recommended dilution for 1 h at room temperature. Then, cells were analyzed by flow cytometry following the manufacturer’s instructions.

### IL-6 *In Vivo* Blockage

Anti-IL6R antibody (Bio X cell) was injected i.p. into mice at a dose of 150 mg/kg. The mice were then treated with ethanol at a dose of 6 g/kg.

### Transcriptome Analysis

Bone marrow-derived cells were cultured with alcohol (0.5%) or vehicle *in vitro* for 5 h. Afterward, total RNA extraction from collected cells was performed according to the manufacturer’s standard instructions (Invitrogen). RNA quality was detected by formaldehyde denaturation electrophoresis and only those samples with ratios approaching 2:1 for the 28S and 18S bands as well as a satisfied RNA integrity number were used. Then samples are sent to agented company (Vazyme Biotech Co., Ltd.) for further transcriptome analysis.

### Assay for Serum Aminotransferase Activity

Serum alanine aminotransferase (ALT) and aspartate aminotransferase (AST) activities were determined using the serum aminotransferase test kit (Biovision US) according to the manufacturer’s instructions and reported in terms of units per liter.

### Q-PCR

Total RNA of sorted G-MDSCs cells was extracted with TRIzol reagent (Invitrogen) and cDNA was synthesized using PrimeScript RT Reagent kit (TaKaRa). Real time PCR was conducted using SYBR Green Master mix (TaKaRa) on Light Cycler 480 PCR system (Roche, USA). The murine β-actin was used as endogenous control. The primer sequences used in this study are listed in Table S2 in Supplementary Material.

### Western Blot

Total protein was extracted from cells or liver tissue *via* RIPA lysis buffer with cocktail proteinase inhibitor and phosphatase inhibitor. The protein lysates were separated on SDS-PAGE gel and transferred to polyvinylidene difluoride membranes. After blocking, the membrane was incubated with corresponding antibody followed by HRP-labeled secondary antibody. The blots were subjected to chemiluminescence analysis.

### Statistical Analysis

Results are expressed as mean ± SD. Comparisons between groups were made by unpaired Student’s *T*-test or two-way ANOVA. *p* < 0.05 was considered statistically significant.

## Results

### CD11b^+^Ly6G^+^ MDSCs Populations Increase in Mice Treated With Ethanol

**Figure 1:**
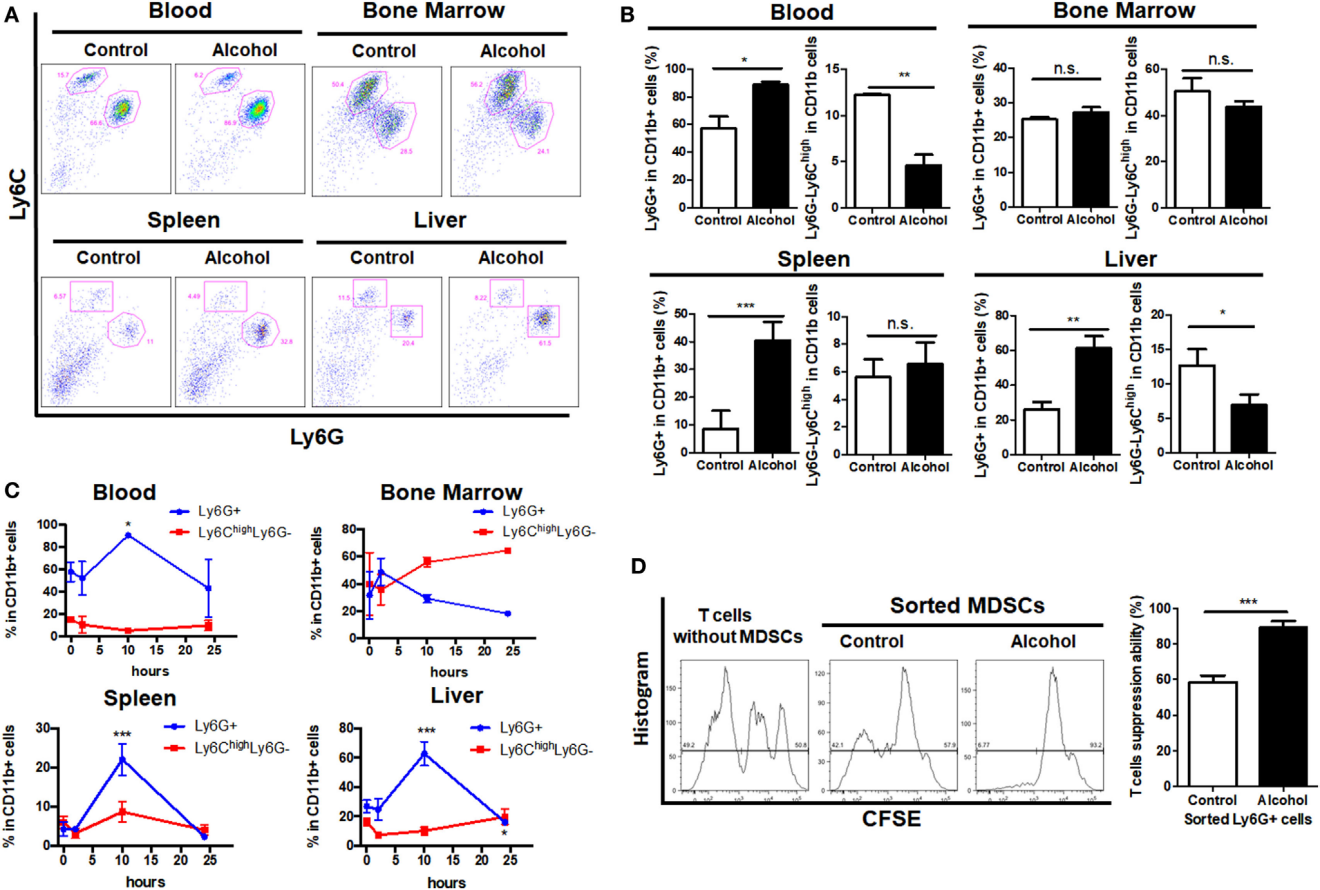
Profile of myeloid-derived suppressor cells (MDSCs) populations in mice treated with vehicle or ethanol. Mice were divided into two groups, ethanol group (*n* = 6) treated with a single dose of ethanol (6 g/kg body weight) *via* gavage and control group (*n* = 6) received isocaloric dextrin-maltose. After 10 h, mice were sacrificed and cells were obtained from blood, liver, spleen, and bone marrow for flow cytometric analyses. **(A)** Representative images and **(B)** quantification of flow cytometric analyses of cells from blood, spleen, liver, and bone marrow of mice with vehicle or ethanol treatment. Then time frame of MDSCs in response to ethanol treatment was elaborated. Mice were sacrificed at 2 h (*n* = 6), 10 h (*n* = 6), and 24 h (*n* = 6) after the ethanol treatment, and populations of granulocytic-MDSCs (G-MDSCs) and monocytic-MDSCs (M-MDSCs) were determined by flow cytometer. **(C)** Time course of G-MDSCs and M-MDSCs in tissues after ethanol exposure. Change of the MDSCs population was normalized within 24 h after a single dose of ethanol. G-MDSCs were sorted from mice treated with vehicle or ethanol by FACS Aria I cytometer (BD) and co-cultured with carboxyfluorescein succinimidyl ester (CFSE)-labeled cytotoxic T cells sorted from spleen of naïve mice. **(D)** Representative histogram images of flow cytometric and quantification analyses of CFSE intensity in CFSE-labeled T cells co-cultured with G-MDSCs cells treated with ethanol or vehicle. Data were analyzed as mean value ± SD and Student’s *T*-test was used to assess the result significance. *p < 0.05, **p < 0.01, ***p < 0.001, compared with the control group; n.s., not significant.

The acute alcoholic liver injury model was successfully established as indicated by histological changes in the liver and significant increased levels of ALT and AST (Figures S1A,B in Supplementary Material). Identification of MDSCs population by gating on the sub-population of G-MDSCs and M-MDSCs was performed (Figure S1C in Supplementary Material). We found that the population of CD11b^+^Ly6G^high^Ly6C^int^ (G-MDSCs) was significantly increased in the blood, spleen, and liver of mice treated with ethanol as compared with controls, while the population of CD11b^+^Ly6C^high^Ly6G^−^ (M-MDSCs) was reduced (Figures 1A,B). This could also be observed in the hematological progenitor population in the bone marrow. The time frame of MDSCs in response to ethanol treatment was elaborated. Increase of G-MDSCs populations and decrease of M-MDSCs were not observable immediately after ethanol consumption, but the population was subsequently changed (Figure 1C). In line with clearance of ethanol, change of the MDSCs sub-population was normalized within 24 h after a single dose of ethanol (Figure 1C). A previous study showed that while neutrophils and MDSCs share a similar profile in cell marker presentation, they have distinct regulatory activities in immune response (36). We isolated the G-MDSCs from mice treated with vehicle or ethanol and co-cultured them with CFSE-labeled cytotoxic T cells. The strategy to analyze and calculate the suppression rate of T cells was shown in Figure S1D in Supplementary Material. Reduced proliferation of the activated T cells revealed that the expanded G-MDSCs populations from ethanol-treated mice present immunosuppressive activity (Figure 1D). The mRNA level of anti-inflammatory cytokine IL-10 was significantly increased in isolated G-MDSCs from the liver of ethanol-treated mice (Figure S1E in Supplementary Material). Moreover, the protein level of IL-10 secreted by G-MDSCs from ethanol-treated mice was significant higher than that of control mice, while the level of GM-CSF was slightly increased without statistic significance (Figure S1F in Supplementary Material). The increased protein level of IL-10 secreted by G-MDSCs from ethanol-treated mice further confirmed that the expanded population is likely to be immunosuppressive MDSCs rather than pro-inflammatory neutrophils. In addition, we found that the immunosuppressive capacity of G-MDSCs was significantly reduced when IL-10R blockade was performed (Figure S1G in Supplementary Material), suggesting that IL-10 might be primarily involved in the immunosuppressive mechanism of this population.

### G-MDSCs Populations Play a Protective Role in Acute Alcoholic Liver Injury

Increasing evidence has highlighted the function of MDSCs in many other pathological conditions such as autoimmunity, infection, and inflammation (16, 35). However, the role of MDSCs in ALD still remains unclear. To examine whether expansion of G-MDSCs population protects the liver from ethanol-induced damage, we applied anti-Gr1 antibody to eliminate the MDSCs population.

**Figure 2:**
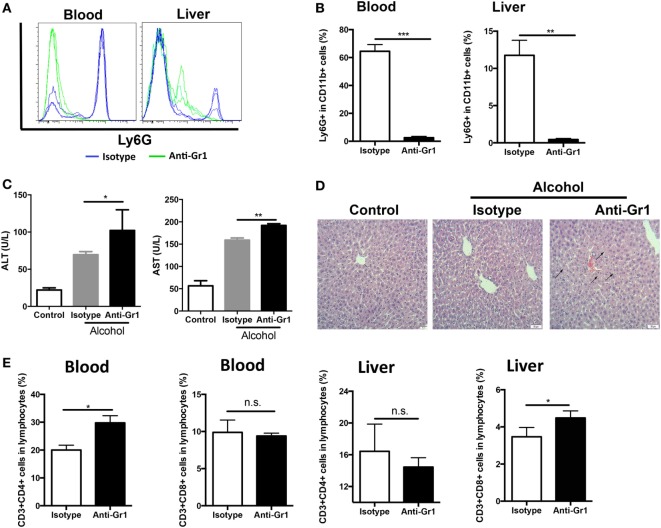
Depletion of granulocytic-MDSCs (G-MDSCs) population aggravated acute alcoholic liver injury. Anti-Gr1 (RB6-8C5) or isotype IgG2b antibody were injected i.p. into mice and then treated with ethanol (*n* = 6 for each group). The populations of myeloid-derived suppressor cells (MDSCs) and T cells in blood and liver were determined by flow cytometer and then liver function was evaluated *via* serological and histological examination. **(A)** Representative histogram images and **(B)** quantification of flow cytometric analyses of G-MDSCs populations in blood and liver of mice treated with anti-Gr1 or vehicle. G-MDSCs cells were effectively depleted following single anti-Gr1 antibody injection. **(C)** The serum alanine aminotransferase (ALT) and aspartate aminotransferase (AST) levels in mice treated with anti-Gr1 were significant higher than that of vehicle. **(D)** The representative H&E staining images of liver tissue from normal mice, ethanol model mice with vehicle injection, and ethanol mice with anti-Gr1 injection. The hepatocellular apoptosis and single cell necrosis were indicated by arrows. **(E)** Population of T helper cells and cytotoxic T cells in blood and liver of model mice and anti-Gr1 treated mice. Data were analyzed as mean value ± SD and Student’s *T*-test was used to assess the result significance. *p < 0.05, **p < 0.01, ***p < 0.001, compared with the control group; n.s., not significant.

CD11b^+^Ly6G^high^Ly6C^int^ G-MDSCs cells were effectively depleted following single anti-Gr1 antibody injection (Figures 2A,B). After anti-Gr1 antibody injection, mice were treated with ethanol and sacrificed to evaluate liver injury. The ALT and AST levels were significantly increased in mice receiving ethanol treatment in the presence of anti-Gr1 antibodies (Figure 2C). Consistently, the histological study also showed more severe liver injury in mice pre-treated with anti-Gr1 than vehicle mice (Figure 2D). Then, we determined the population of T cells in tissues after G-MDSCs depletion and found that T helper cells were significantly increased in blood, and cytotoxic T cells were also raised in the liver (Figure 2E). The results indicated that the severity of alcoholic liver injury was increased by anti-Gr1 antibody depletion, suggesting the cytoprotective role of G-MDSCs cells, especially the CD11b^+^Ly6G^high^Ly6C^int^ population in acute alcoholic liver injury.

**Figure 3:**
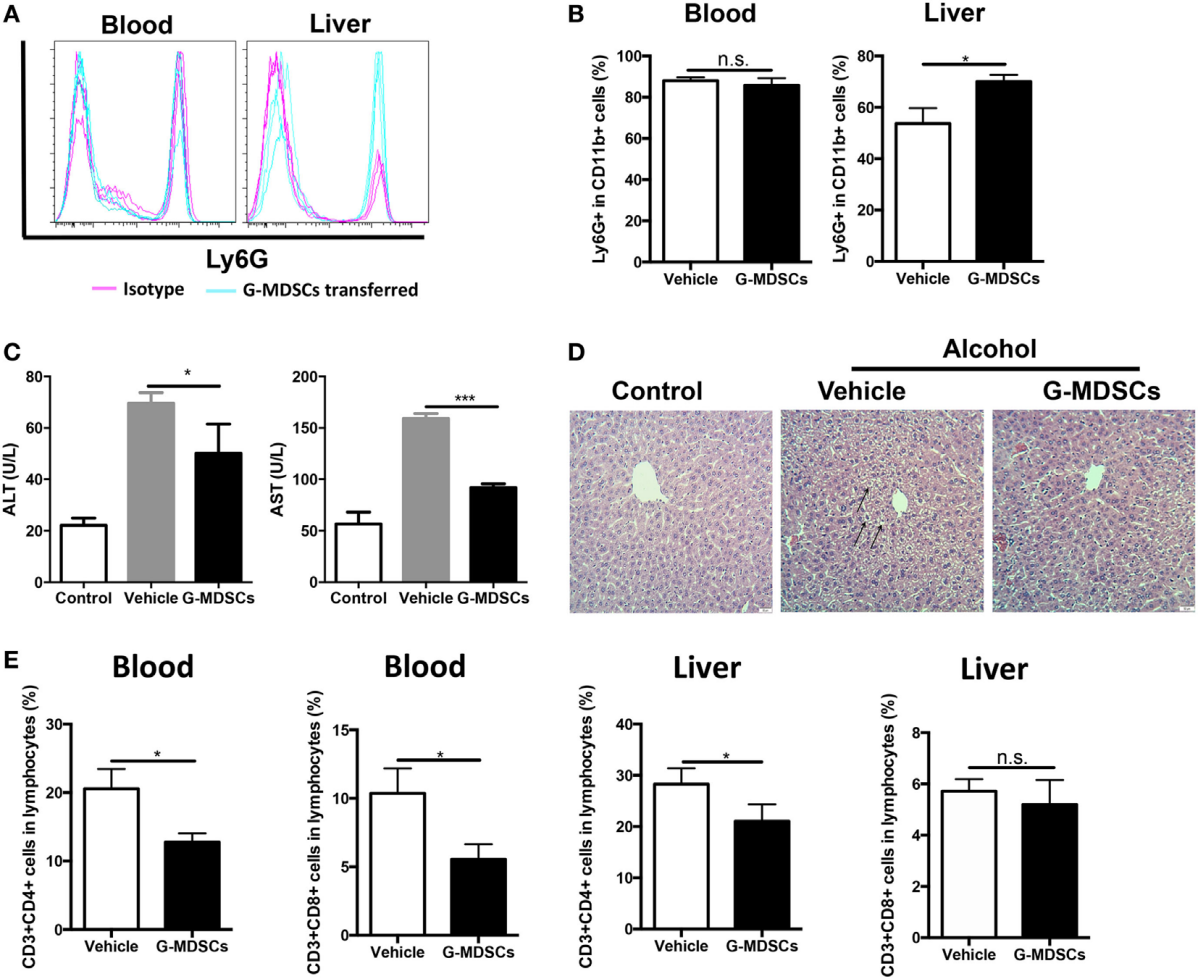
Adoptive transfer of granulocytic-MDSCs (G-MDSCs) populations alleviated liver injury induced by ethanol. About 1 × 10^7^ CD11b^+^Ly6G^high^Ly6C^int^ cells with over 90% purity in 200 µL PBS were injected into naïve mice before receiving ethanol (*n* = 5). The populations of myeloid-derived suppressor cells (MDSCs) and T cells in blood and liver were determined by flow cytometer and then liver function was evaluated *via* serological and histological examination. **(A)** Representative histogram images and **(B)** quantification of flow cytometric analyses of G-MDSCs populations in blood and liver of mice receiving PBS or transferred G-MDSCs. The population of G-MDSCs was significantly increased in the liver after G-MDSCs adoptive transfer, while their percentage was comparable in blood. **(C)** Alanine aminotransferase (ALT) and aspartate aminotransferase (AST) levels in serum of normal mice, ethanol model mice with vehicle injection, and ethanol mice with G-MDSCs transferred. **(D)** The representative H&E staining images of liver tissue from normal mice, ethanol model mice with vehicle injection, and ethanol mice with G-MDSCs transferred. The hepatocellular apoptosis and single cell necrosis were indicated by arrows. **(E)** Population of T helper cells and cytotoxic T cells in the blood and liver of model mice and G-MDSCs-treated mice. Data were analyzed as mean value ± SD and Student’s *T*-test was used to assess the result significance. *p < 0.05, ***p < 0.001, compared with the control group; n.s., not significant.

Although anti-Gr1 antibody has been extensively used to eliminate MDSCs in animal models, antibody depletion alone may not be adequate to define the role of G-MDSCs in the pathogenesis of acute alcoholic liver injury, as Gr1 is also expressed in other cells like CD11b^−^Gr1^+^ granulocytes, despite in a small proportion. To strictly and comprehensively examine the contribution of MDSCs, we further carried out adoptive transfer study of G-MDSCs populations. About 1 × 10^7^ CD11b^+^Ly6G^high^Ly6C^int^ cells with over 90% purity in 200 µL PBS were injected into naïve mice before receiving ethanol. Compared with PBS injection group, the population of CD11b^+^Ly6G^high^Ly6C^int^ cells was significantly increased in the liver after G-MDSCs adoptive transfer, while their percentage was comparable in blood (Figures 3A,B), spleen, and bone marrow (data not shown). The results showed that the transferred G-MDSCs populations predominantly homed to the inflammatory site (Figures 3A,B), which is consistent with previous studies (29, 35). To further clarify that this population was G-MDSCs cells rather than pro-inflammatory neutrophils, we sorted this population and then co-cultured with T cells *in vitro* to evaluate its immune suppressive capacity. The results indicated that this population could suppress the proliferation of T cells (Figure S2A in Supplementary Material). In addition, the mRNA expression levels of arginase-1, GM-CSF, and IL-10 of this population were increased (Figure S2B in Supplementary Material). The protein levels of GM-CSF and IL-10 were significantly enhanced in the sorted G-MDSCs from liver of transferred mice compared to that of control mice (Figure S2C in Supplementary Material). The above results indicated that these increased CD11b^+^Ly6G^high^Ly6C^int^ cells in the liver were G-MDSCs populations but not pro-inflammatory neutrophils. In line with the results from the G-MDSCs depletion study, adoptive transfer of G-MDSCs alleviated ethanol-induced liver injury, which is demonstrated by lowered ALT and AST levels as well as improved histological changes in the liver (Figures 3C,D). It was found that both T helper cells and cytotoxic T cells were decreased significantly in the blood of mice with transferred G-MDSCs cells (Figure 3E). In addition, in the liver of mice receiving G-MDSCs cells, T helper cells were also found to be significantly decreased (Figure 3E). T-cell adoptive transfer was performed to study the role of T cells in mediating alcoholic liver injury. We found that the levels of ALT and AST were slightly increased in the T-cell transferred group compared with the control group, although this difference was not statistically significant (Figure S3 in Supplementary Material). Moreover, the concentration of serum TNF-α was also significantly decreased in the G-MDSCs cell transferred group and increased in the G-MDSCs cell depletion group compared to that of vehicle-treated mice (Figure S4 in Supplementary Material). In summary, according to the above results from loss- and gain-of-function analyses, we conclude that the expanding G-MDSCs played a cytoprotective role in mediating ethanol-induced acute liver injury.

### Ethanol Promotes the Preferential Generation of G-MDSCs Populations From Myeloid Precursors *via* Chemokine Interaction and YAP/Hippo Signaling *In Vitro*

**Figure 4:**
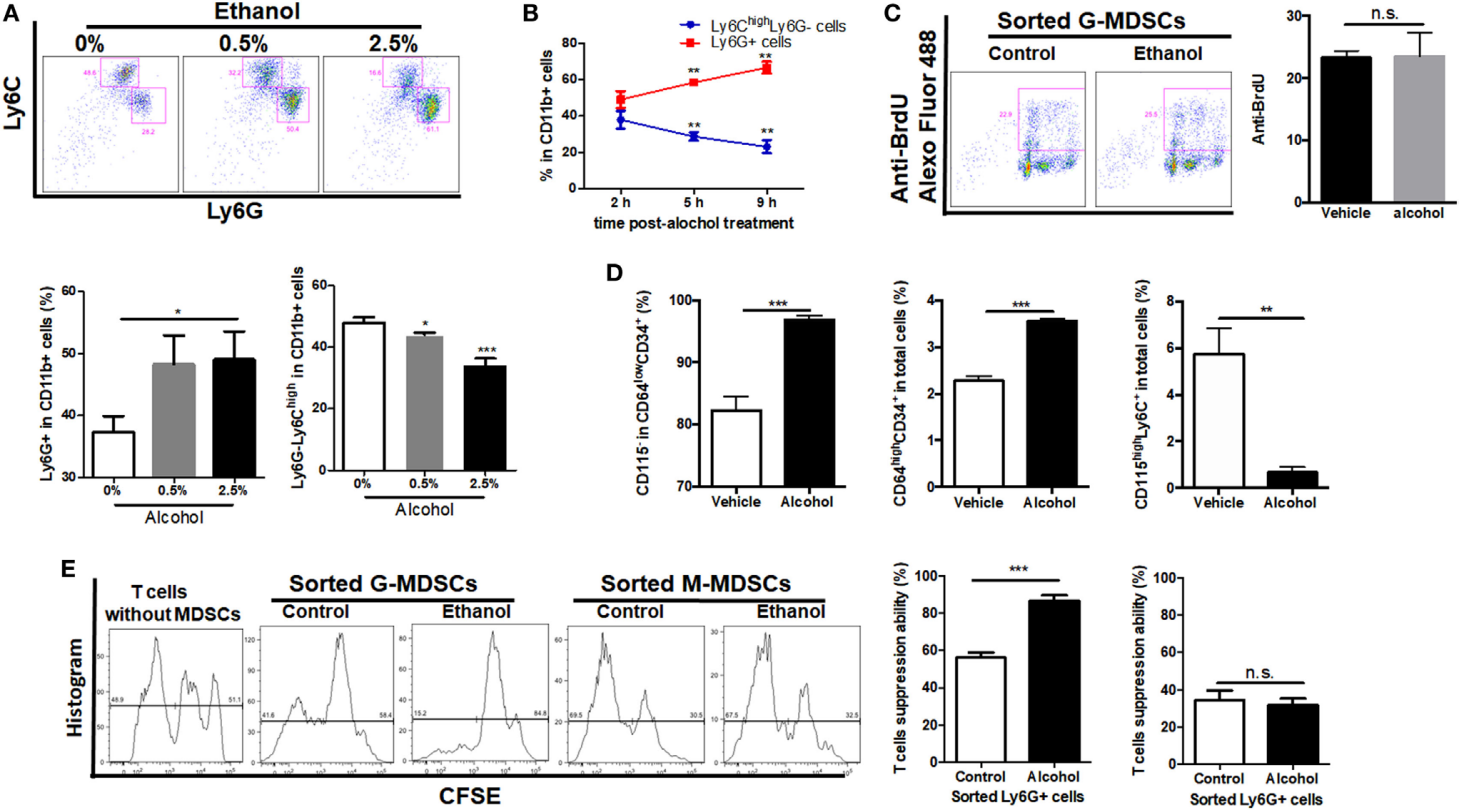
Effect of ethanol on the differentiation, expansion, and function of myeloid-derived suppressor cells (MDSCs) populations *in vitro*. Bone marrow-derived cells were isolated from naïve mice and treated directly with 0.5 or 2.5% ethanol in RPMI-1640 medium supplemented with 10% FBS. After culturing for 2, 5, and 9 h, cells were collected for the examination of MDSCs population *via* flow cytometer. **(A)** Representative images and quantification of flow cytometric analyses granulocytic-MDSCs (G-MDSCs) populations treated with ethanol at different concentrations. **(B)** Time course of population of G-MDSCs and monocytic-MDSCs (M-MDSCs) cultured with ethanol at the concentration of 0.5% (vol/vol). **(C)** Representative images and quantification of flow cytometric analyses anti-BrdU Alexo Flour 488 staining. Bone marrow-derived cells were labeled with BrdU at a final concentration of 10 µM. Then, cells were analyzed by flow cytometer. The proliferation of G-MDSCs was not increased by ethanol treatment. **(D)** Populations of common myeloid precursor, granulocyte-monocyte progenitors (GMPs), and monocyte-committed progenitors produced by GMPs (MP) in bone marrow-derived cells treated with ethanol or vehicle determined by flow cytometer. **(E)** Representative images of flow cytometric analyses of carboxyfluorescein succinimidyl ester (CFSE) intensity in CFSE-CD8^+^ T cells co-cultured with G-MDSCs populations or M-MDSCs treated with ethanol or vehicle. Data were analyzed as mean value ± SD and Student’s *T*-test was used to assess the result significance. *p < 0.05, **p < 0.01, ***p < 0.001, compared with the control group; n.s., not significant.

As ethanol can freely diffuse into the circulating system, it can interact with the hematological cell lineage and has direct regulation on the expansion, differentiation, and function of MDSCs. Bone marrow-derived cells were isolated and treated directly with 0.5 or 2.5% ethanol. It was shown that ethanol treatment directly induced expansion of G-MDSCs populations, while reduced M-MDSCs in a dose-dependent manner (Figure 4A). The effect of ethanol on MDSCs population was eliminated after 12 h of ethanol stimulation (data not shown), which was consistent with our *in vivo* observations. Since ethanol impacts on G-MDSCs significantly at 0.5% (vol/vol), the time frame of ethanol-treated G-MDSCs profiling was also studied at this concentration. The population of G-MDSCs was significantly increased while M-MDSCs were significantly decreased compared with the control group (Figure 4B). The results indicated that ethanol *per se* favors the differentiation of G-MDSCs population. The underlying mechanism might be by inducing the proliferation of G-MDSCs populations or facilitating the preferential generation of G-MDSCs from myeloid precursors or promoting the differentiation from M-MDSCs to G-MDSCs populations. To evaluate the potential mechanism, we first performed anti-BrdU-staining to study the proliferation of G-MDSCs in ethanol-treated animals. However, results showed that the proliferation of G-MDSCs was not increased by ethanol treatment (Figure 4C). Then, we further determined the population change of common myeloid precursor (CMP) CD64^low^CD34^+^CD115^−^ as well as granulocyte-monocyte progenitors (GMPs) CD64^high^CD34^+^ treated with ethanol (37, 38). The results showed that populations of CD64^low^CD34^+^CD115^−^ and CD64^high^CD34^+^ were significantly increased, while the CD115^high^Ly6C^+^ population and monocyte-committed progenitors produced by GMPs (named MP) were significantly decreased by ethanol treatment (Figure 4D). This hints that ethanol could affect the differentiation of these precursors or progenitors. In particular, it could promote the generation of CMP and GMPs, whereas it could impede the differentiation from GMPs to MP, suggesting more precursors preferentially generate G-MDSCs. To examine if direct stimulation of ethanol could promote the immunosuppressive function of MDSCs, we co-cultured the ethanol-treated G-MDSCs and M-MDSCs from bone marrow with primed T cells isolated from mouse spleen. CSFE-labeled T cells when in contact with ethanol-treated G-MDSCs showed lower proliferation rate, indicating that direct stimulation of ethanol provokes the immune suppressive effect of G-MDSCs populations (Figure 4E). Regarding M-MDSCs, its immune suppressive function on T cells was not as strong as G-MDSCs populations, and we found that ethanol could not increase its immune suppressive capacity on T cells (Figure 4E).

**Figure 5:**
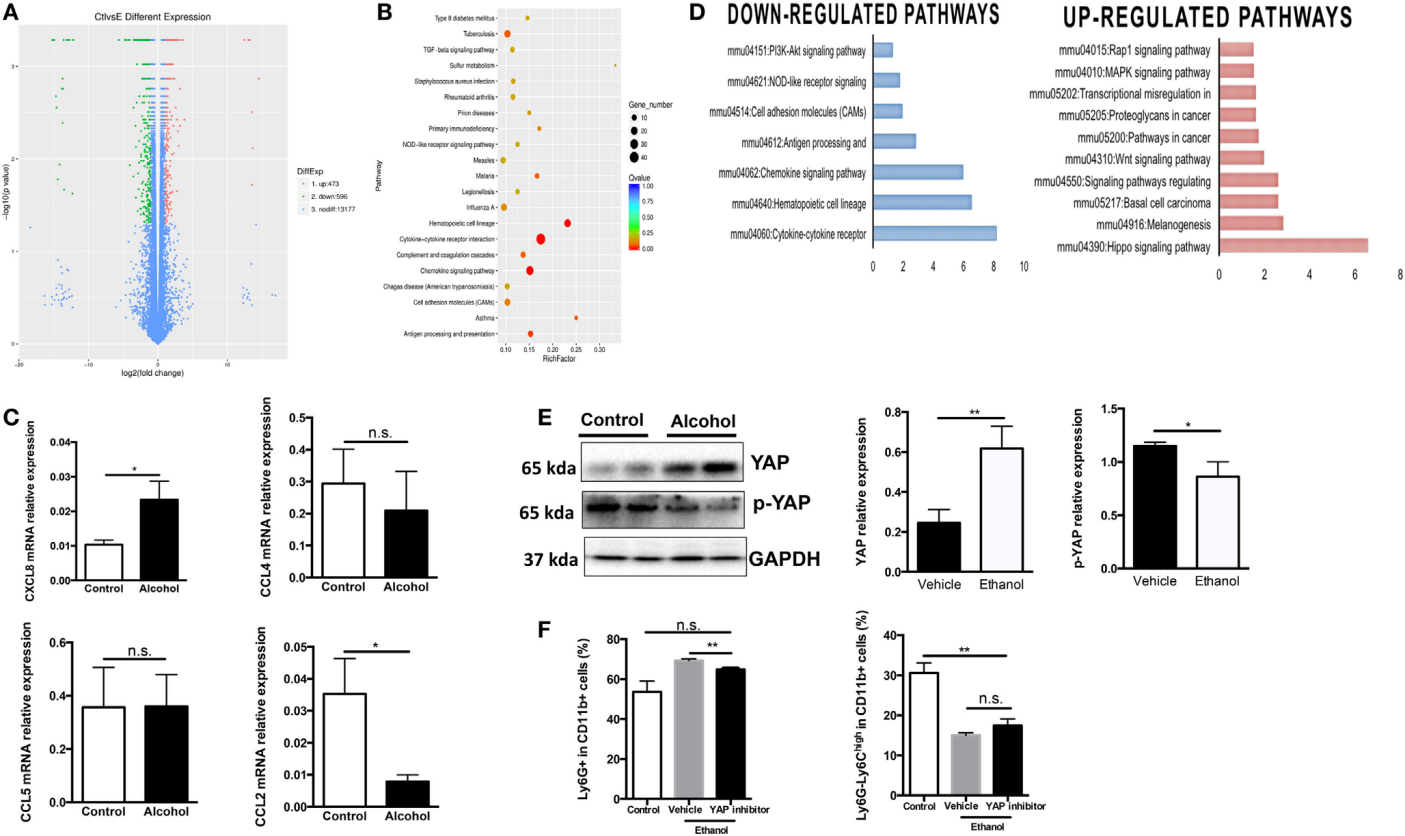
Molecular mechanism underlying ethanol-stimulated granulocytic-MDSCs (G-MDSCs) expansion and functioning. Bone marrow-derived cells treated with alcohol or vehicle for 5 h *in vitro* and subjected to transcriptomic analysis (*n* = 3 for each group). **(A)** Volcano plot of differentially expressed genes in cells treated with PBS or ethanol. **(B)** Gene ontology analysis. **(C)** The mRNA level of several chemokine in sorted G-MDSCs cells treated with ethanol or vehicle *in vitro* determined by q-PCR. **(D)** Related downregulated pathways and upregulated pathways by ethanol treatment. **(E)** The protein expression level and phosphorylation level of YAP detected by western blotting in sorted G-MDSCs cells treated with ethanol or vehicle. Total protein was extracted from sorted G-MDSCs treated with vehicle or ethanol (*n* = 4 for each group). **(F)** Presence of YAP inhibitor abrogates G-MDSCs expansion induced by ethanol. YAP inhibitor verteporfin was added into the culture medium at the final concentrations 2 µg/mL. The population of G-MDSCs was determined after culturing 5 h with ethanol. Data were analyzed as mean value ± SD and Student’s *T*-test was used to assess the result significance. *p < 0.05, **p < 0.01, compared with the control group; n.s., not significant.

To better understand the molecular mechanism underlying ethanol-stimulated G-MDSCs expansion and functioning, we applied transcriptomic analysis to reveal possible differential gene expression in hematological cell lineage after ethanol treatment. A series of gene expression was changed by ethanol treatment (Figure 5A). Gene ontology analysis was conducted and the biological process with significance was enriched (Figure 5B). It was seen that ethanol regulates the biological process related to hematopoietic cell lineage and cytokine/chemokine-receptor interaction, which was in line with our experimental observations. The mRNA levels of several chemokine such as CXCL8, CCL2, CCL4, and CCL5 in sorted G-MDSCs cells treated with ethanol or vehicle *in vitro* were detected, and it was found that CXCL8 was increased significantly, while CCL2 was decreased significantly after ethanol treatment for 5 h (Figure 5C). Further pathway analysis enrichment showed that the Hippo signaling pathway was the most significantly upregulated one (Figure 5D). We also found that ethanol stimulation activated YAP in G-MDSCs, which is the core molecule-mediated activation of Hippo signaling pathway (Figure 5E). The phosphorylation of YAP was significantly decreased by ethanol treatment (Figure 5E). Addition of YAP inhibitor abrogated the effect of ethanol on G-MDSCs population (Figure 5F). Hyperactivated Hippo-YAP signaling in driving upregulation of CXCL5 *via* the YAP–TEAD complex and stimulating MDSC recruitment have been identified in cancer cells (39). YAP1 directly regulates *Cxcl5* transcription, a ligand for CXCR2-expressing MDSCs, thereby facilitating MDSCs recruitment. In the present study, we found CXCL8 was upregulated by ethanol (Figure 5C). Taken together, these observations suggest free diffusion of ethanol into the circulating system after consumption directly regulated the functions of G-MDSCs, which may be related to the activation of Hippo-YAP signaling and chemokine interaction.

### Role of IL-6 in Mediating Expansion of G-MDSCs

**Figure 6:**
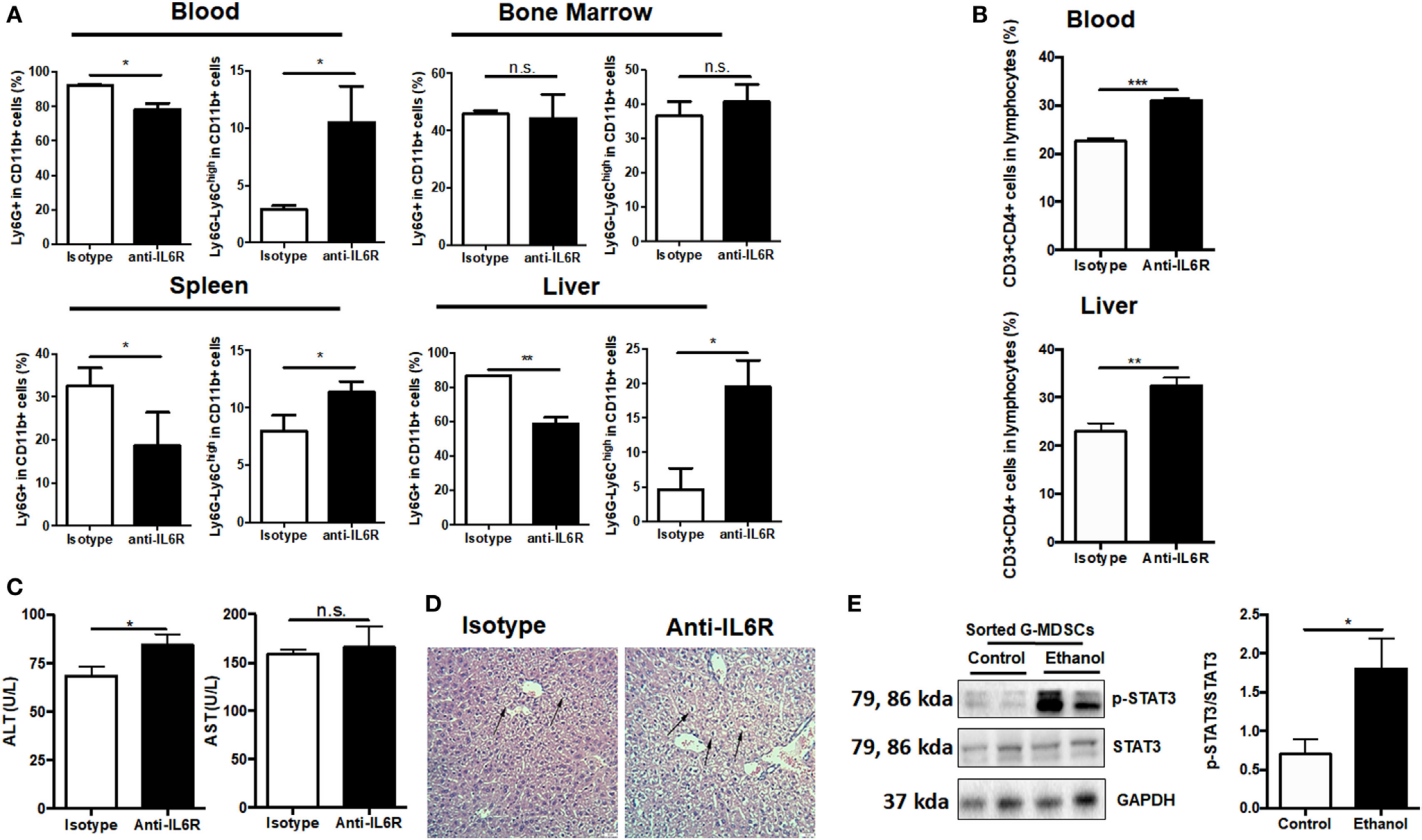
Role of IL-6 in mediating the expansion of granulocytic-MDSCs (G-MDSCs) populations. Anti-IL6R antibody (Bio X cell) or isotype antibody was injected i.p. into mice at a dose of 150 mg/kg per mouse (*n* = 6 for each group). The populations of myeloid-derived suppressor cells (MDSCs) and T cells in blood and liver were determined by flow cytometer and then liver function was evaluated *via* serological and histological examination. **(A)** Population of MDSCs from the blood, spleen, liver, and bone marrow of mice treated with anti-IL6R or isotype antibody. **(B)** Population of cytotoxic T cells and T helper cells in blood and liver of mice treated with anti-IL6R or isotype antibody. **(C)** Alanine aminotransferase (ALT) and aspartate aminotransferase (AST) level in serum of mice treated with anti-IL6R or isotype antibody. **(D)** H&E staining images of liver tissues from mice treated with anti-IL6R or isotype antibody. The hepatocellular apoptosis and single cell necrosis were indicated by arrows. **(E)** Expression of p-signal transducer and activator of transcription-3 (STAT3), and STAT3 in G-MDSCs sorted from mice treated with ethanol or vehicle detected by western blotting. Data were analyzed as mean value ± SD and Student’s *T*-test was used to assess the result significance. *p < 0.05, **p < 0.01, ***p < 0.001, compared with the control group; n.s., not significant.

As IL-6 has been intensively implicated in inducing MDSCs (40, 41) and we observed increased IL-6 content in the serum after ethanol administration (Figure S5A in Supplementary Material), we speculated that IL-6 might play a role in mediating the expansion of G-MDSCs in acute alcoholic liver injury. First, anti-IL6R antibody was used to block IL-6 *in vivo*. We found that ethanol-mediated expansion of G-MDSCs was significantly reduced by anti-IL6R injection (Figure 6A). Furthermore, the population of M-MDSCs was remarkably raised in the blood, liver, and spleen of anti-IL6R injection group (Figure 6A). The change of MDSCs population in the bone marrow was not significant (Figure 6A). Meanwhile, the content of T helper cells was consistently increased in the blood and liver (Figure 6B). We further evaluated the severity of liver injury. The significant increase of ALT level and worsening histological performance indicated that the liver injury induced by ethanol was more severe after anti-IL6R treatment (Figures 6C,D). The ethanol-increased IL-6 binds to IL-6 receptor expressed on G-MDSCs cells surface, thereby activating the expansion pathway. We confirmed the function of IL-6 in our *in vitro* study and found that the population of G-MDSCs was increased by IL-6 treatment while M-MDSCs were decreased significantly (Figure S5B in Supplementary Material). It was identified that IL-6 signaling resulted in the phosphorylation of the signal transducer and activator of transcription-3 (STAT3) that was involved in the accumulation of MDSCs (41–43). Accordingly, the level of p-STAT3 in isolated G-MDSCs from the liver of mice treated with ethanol was significantly increased compared to the control group (Figure 6E). Moreover, we observed that the mRNA level of S100A8 in G-MDSCs from the liver and blood of mice treated with ethanol was also significantly improved (Figure S5C in Supplementary Material). Meanwhile, the expression of S100A8 was also significantly increased in G-MDSCs cultured with ethanol *in vitro* (Figure S5D in Supplementary Material). The correlation analysis between the amounts of pSTAT3 and S100A8 in isolated G-MDSCs from the liver of mice has been performed. The fold changes of pSTAT3/STAT3 are positively correlated with the amount of S100A8 (*r*^2^ = 0.8507) (Figure S5E in Supplementary Material). S100A8 serves as the down-stream target of activated STAT3 to promote the accumulation of MDSCs (44). Thus, IL6–STAT3–S100A8 might be involved in the process of G-MDSCs cell expansion after ethanol exposure.

## Discussion

Myeloid-derived suppressor cells are a heterogeneous population with Gr1 antigen expression, which can be further divided into two major subsets: G-MDSCs and M-MDSCs (15). The similar phenotypes and surface markers shared by G-MDSCs and neutrophils make it difficult to distinguish them, which are actually also one of challenges in this field (32). In mice, several parameters that can distinguish G-MDSCs from polymorphonuclear cells have been suggested; however, none is sufficient, and more effort is needed to better distinguish these cells (37). There are no specific markers available currently to separate mature neutrophils from the population of G-MDSCs. In the gated CD11b^+^Ly6G^high^Ly6C^int^ population, certain mature neutrophils are inevitably included. However, in our study, we also proved that this population possesses some MDSCs function such as T-cell suppression. Therefore, we defined this CD11b^+^Ly6G^high^Ly6C^int^ population as G-MDSCs in this setting.

Recently, MDSCs have been studied in acute liver inflammation and are usually associated with beneficial functions (45–48). However, controversy about which subsets are preferentially involved in different pathological settings has been raised (49). It has been proposed that M-MDSCs might be the responsible subset with immune suppressive capacity to alleviate liver damage in several inflammatory models. However, in the present study, we found that it was the G-MDSCs populations rather than M-MDSCs that expanded significantly in blood, spleen, and liver after acute ethanol exposure. In most of the tumor models, a preferential expansion of G-MDSCs populations has also been demonstrated (50). Regarding the precursor-progeny link between these two myeloid cell subsets, GMPs might be the precursors of G-MDSCs populations, while M-MDSC might differ from MP (37, 38). We found that ethanol could affect the differentiation of these precursors or progenitors. In particular, it could promote the generation of CMP and GMPs and impede the differentiation from GMPs to MP, suggesting more precursors preferentially generate G-MDSCs populations. It has been proposed that GMPs might also commit the generation of M-MDSCs (51). The increased M-MDSCs would further convert into G-MDSCs by an epigenetic mechanism, resulting the expansion of G-MDSCs. We further revealed that the expansion of G-MDSCs significantly contributes to attenuation of alcoholic liver injury by loss- and gain-of-function analysis. Currently, an anti-Gr1-based approach is always an option to eliminate MDSCs *in vivo* despite being controversial because better alternatives such as genetic knockout or knockdown are unavailable. The depletion efficiency has been well documented in many studies, whereas it was also reported that anti-Gr1 antibody failed to eliminate MDSCs in the liver (52–56). The discrepancies might be attributed to different models or regimens of antibodies used in these studies. In our study, G-MDSCs were effectively depleted following single anti-Gr1 antibody injection. Another concerning issue is that Gr1 antibody may deplete other cells expressing Gr1 such as Gr1^+^CD11b^−^ granulocytes. As over 90% Gr1^+^ cells in the liver and circulation of mice are MDSCs, the conclusion we drew from anti-Gr1 depletion is still valid (29). It cannot be denied that the depletion of other Gr1 expressing cells might have an uncertain influence on the disease; therefore, adoptive transfer study is necessary to comprehensively define the role of G-MDSCs. After G-MDSCs transfer, the population of G-MDSCs was significantly increased in the liver of mice treated with ethanol, but not in the blood, spleen, or bone marrow. Our findings support the claim that exogenous MDSCs home in on the inflamed site. Furthermore, we have found that in mice with three consecutive ethanol exposures, the increase of G-MDSCs population was not observed as much in mice having one excessive ethanol exposure. As we cannot yet fully explain the underlying mechanism, data are not shown here and merit further investigation. The expanding and infiltrating G-MDSCs populations might be the body’s attempt to relieve ethanol-induced injuries. However, once the stimulation is overwhelming, this self-protection might be turned off gradually, leading to persistent inflammation and tissue injury. As a matter of fact, a complicated role was indicated from available limited data on the involvement of MDSCs in chronic liver injury (49). Both beneficial and detrimental roles of MDSCs in the setting of chronic hepatofibrogenesis have been revealed (30, 57, 58). On the one hand, this tolerogenic mechanism may limit immune responses and subsequent hepatic damage, but on the other hand, immune tolerance may inhibit pathogen eradication and facilitate chronic infections (49). Thus, more data are needed to clarify the involvement of MDSCs in chronic hepatic inflammatory settings.

Compelling evidence indicates that both innate and adaptive immunities triggered by ethanol-induced oxidative modifications of hepatic constituents contribute to the progression of ALD (1, 59, 60). Although innate immunity is considered to play a central role, increasing studies have also reported that cytotoxic T cells and T helper cells infiltrate into the liver in alcoholic hepatitis and active alcoholic cirrhosis, thus leading to the production of pro-inflammatory cytokines like TNF-α (59, 60). In the present study, we observed that both CD8^+^ cytotoxic T cells and CD4^+^ T helper cells were significantly increased in the liver after ethanol exposure, which are targeted by G-MDSCs. The level of TNF-α was significantly increased by anti-Gr1 administration while it was decreased significantly after G-MDSCs transfer. Correspondingly, liver injury induced by ethanol was aggravated after G-MDSCs depletion and was alleviated by G-MDSCs adoptive transfer. Therefore, we concluded that the expanding G-MDSCs cells might protect the liver from alcoholic injury through cytotoxic T cells and T helper cells suppression, thus inhibiting inflammatory mediators like TNF-α.

A variety of factors have been involved in the expansion of MDSCs. Factors that have been reported to mediate the expansion of MDSCs include GM-CSF, macrophage colony-stimulating factor, granulocyte-stimulating factor, stem-cell factor, cyclooxygenase 2, vascular endothelial growth factor, IL-6, IL-1β, and TNF-α. Of note, the JAK/STAT pathway activated by factors such as IL-6 has a vital role in mediating both the expansion of MDSCs and their immune suppressive function. STAT3 mediates the expansion and accumulation of MDSCs mainly by stimulating myelopoiesis and inhibiting differentiation of immature myeloid cells into dendritic cells and macrophages *via* upregulation of S100A8/9, and it fosters survival of MDSCs by inducing the expression of MYC, BCL-XL, and cyclin D1. IL-6 is a pleiotropic cytokine that is implicated in both pro- and anti-inflammation (61). The elevated IL-6 in immune-mediated liver disease has been suggested to protect the liver from injury (41). In the present study, we found that the content of IL-6 was significantly elevated in the serum of mice treated with ethanol. As IL-6 is a well-known factor involved in the expansion of MDSCs, we hypothesized that ethanol might regulate the expansion of MDSCs *via* IL-6 production. To block the effect of IL-6 *in vivo*, anti-IL6R antibody was applied as both soluble and membrane IL6R could be restrained. As expected, the population of G-MDSCs was significantly lowered after anti-IL6R treatment, and the alcoholic liver injury was aggravating. After anti-IL6R antibody treatment, we found that the percentage of G-MDSCs population was decreased while M-MDSCs increased in the blood, liver, and spleen. As IL-6 has been demonstrated to be involved in the induction of G-MDSCs, the expansion of M-MDSCs might be due to anti-IL6R impeding the differentiation of GMPs from G-MDSCs cells. The increased GMPs induced by ethanol treatment might be inclined to differentiate into monocytes, resulting in the expansion of M-MDSCs. Our data indicated that IL-6 is partially responsible for the expansion of G-MDSCs populations in alcoholic liver injury. Next, we focused on exploring where the elevated IL-6 comes from. Many cells including liver cells, neutrophils, T cells, and macrophages can secrete IL-6 to stimulate immune response (61). As for MDSCs, on one hand, IL-6 receptors are expressed on the surface of MDSCs; on the other hand, MDSCs can also secrete IL-6 *per se* (62, 63). First, the mRNA expression of IL-6 in the liver treated with ethanol was significantly increased. We also found that the mRNA expression of IL-6 was significantly increased in the sorted MDSCs from mice treated with ethanol. Thus, we propose that the IL-6 secreted by activated T cells and liver cells leads to the increase of MDSCs, and then IL-6 might also serve as a positive self-loop in the expanding MDSCs. However, we also noticed that anti-IL6R treatment could not totally block the expansion of G-MDSCs populations, suggesting that additional mediators might be involved in inducing the expansion of MDSCs cells. It was discovered that the expression of S100A8 was significantly increased in MDSCs isolated from mice with ethanol exposure. S100A8 is a member of the S100 family of calcium-binding proteins released by myeloid origin cells. It has been reported that the S100 family of inflammatory mediators serve as an autocrine feedback loop that maintains expansion of MDSCs (44). The expression of S100A8 was significantly increased, whereas no significant increase of IL-6 expression was determined in the bone marrow-derived cells cultured with ethanol *in vitro*. The expansion of G-MDSCs *in vitro* might be partially attributed to the increased S100A8. Therefore, both IL-6 and S100A8 are implicated in the process of G-MDSCs expansion induced by ethanol. In summary, our data indicated that the increase of G-MDSCs populations partially mediated by IL-6 and S100A8 protects the liver from injury at the early phase of ALD through inhibiting the T-cell response and thus reducing the action of inflammatory mediators like TNF-α. Although this subset has not been clearly identified in the present study owing to the lack of a more specific marker, our research nevertheless contributes to better understanding of the role of immune suppression in acute alcoholic liver injury and reinforces the idea that G-MDSCs might be an attempt of the body to diminish the injurious effects of prolonged inflammation by turning off the effector T lymphocytes under inflammatory conditions (64). In our study, experiments are based on murine model, translational data of humans exposed to alcohol is extremely important to a full and thorough understanding of role of G-MDSCs in alcoholic liver injury. Nevertheless, our findings from mouse model experiments might be relevant to the vulnerability and susceptibility of alcoholic liver injury of individual human beings. The extent of expansion of G-MDSCs might be served as indicator for alcohol tolerance of alcoholics.

## Conclusion

Our study has identified the role and regulation of G-MDSCs in response to acute alcoholic beverage consumption. G-MDSCs were expanded in response to ethanol while M-MDSCs were reduced. The increase of G-MDSCs enhances its immunosuppressive activities by inhibiting T-cell proliferation and pro-inflammatory cytokine expression. Deletion of G-MDSCs exacerbates liver injury caused by ethanol while adoptive transfer of G-MDSCs populations prevents liver damage. Ethanol directly regulates expansion and function of G-MDSCs by activating YAP signaling, and IL-6 is a dominant indirect factor that mediates the induction of G-MDSCs population by acute ethanol consumption. These findings provide new insight into the modulation of the immune system upon acute alcoholic liver injury.

## Ethics Statement

This study was carried out in accordance with the recommendations of Guidelines for the Use of Experimental Animals, the Committee on the Use of Live Animals in Teaching and Research (CULATR) of The University of Hong Kong, Hong Kong. The protocol was approved by the Committee on the Use of Live Animals in Teaching and Research (CULATR) of The University of Hong Kong, Hong Kong (document number: 3646-15).

## Author Contributions

YF conceived the idea, designed the experimental plan, and revised the whole manuscript. SL performed the experiments and drafted the manuscript. NW contributed toward study design, experimental setup, results supervision, and manuscript correction. H-YT, MH, HL, and M-FY contributed to interpret the data and revise manuscript. All authors discussed the results and commented on the manuscript.

## Conflict of Interest Statement

The authors declare that the research was conducted in the absence of any commercial or financial relationships that could be construed as a potential conflict of interest.
