# Supplementary material for: Expansion of Granulocytic, Myeloid-Derived Suppressor Cells in Response to Ethanol-Induced Acute Liver Damage
Source: Front Immunol. 2018 Jul 19;9:1524. doi: 10.3389/fimmu.2018.01524 (PMC6060237; doi:10.3389/fimmu.2018.01524)
Supplement: Supplementary file 1 [file Data_Sheet_1.PDF]

Supplemental Figures

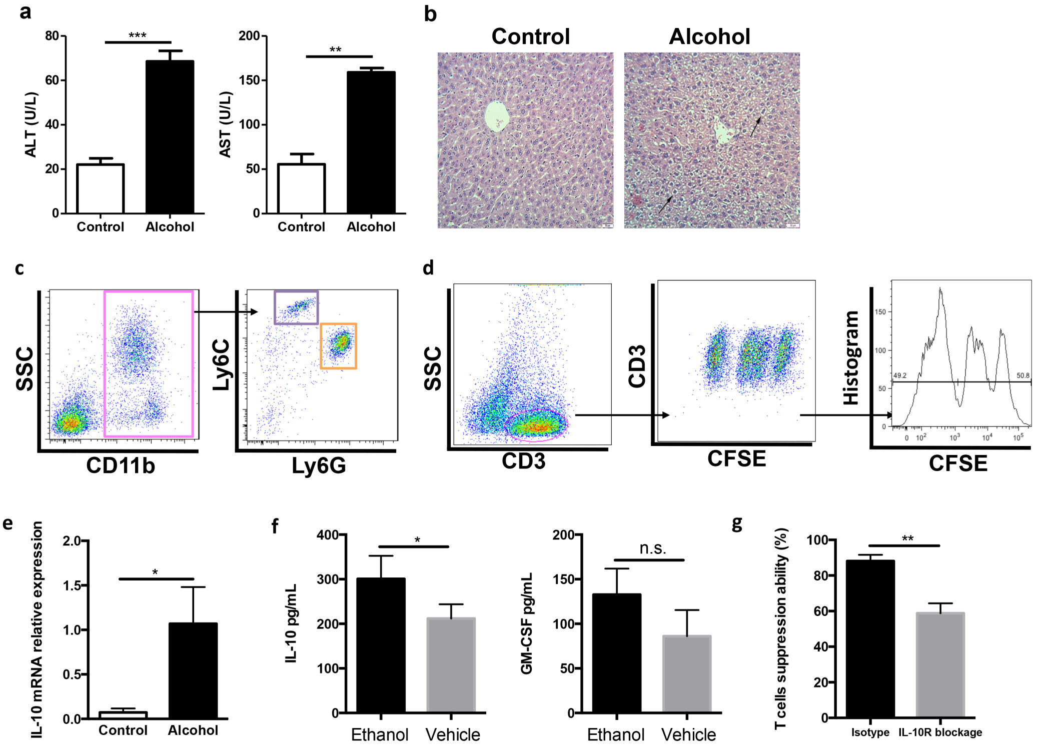

Fig. S1. a), ALT and AST level of control mice or mice treated with alcohol (n=6). b), Representative H&E staining images of liver tissue from normal mice and ethanol model mice. The hepatocellular apoptosis and single cell necrosis were indicated by arrows. c), Strategy to gate G-MDSCs and M-MDSCs population. d), Strategy to gate and calculate the suppression ratio of the proliferation of T cells. e), The mRNA level of IL-10 in isolated G-MDSCs from mice treated with vehicle or ethanol (n=6) determined by q-PCR. f), The protein levels of IL-10 and GM-CSF in isolated G-MDSCs from mice treated with vehicle or ethanol determined by LEGENDplex™ Mouse Inflammation Panel (13-plex) (n=6). g), Representative images of flow cytometric and quantification analyses of CFSE intensity in CFSE-labeled T cells co-cultured with G-MDSCs from mice treated with ethanol in presence of anti-IL-10R or isotype antibody. Data were analyzed as mean value ± SD and Student's T-test was used to assess the result significance. *p<0.05, **p<0.01, ***p<0.001, compared with the control group; n.s., not significant.

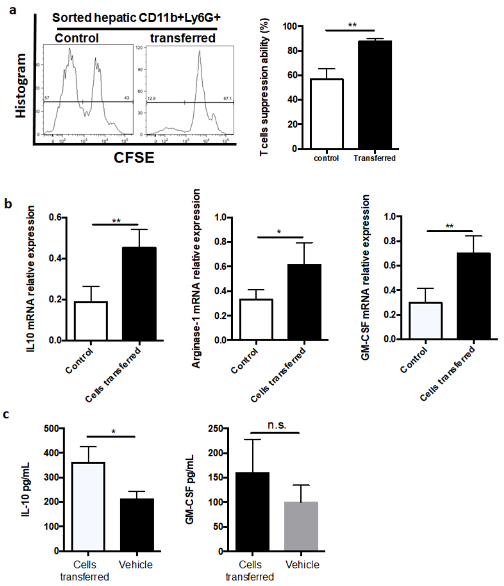

Fig. S2. a), Representative images of flow cytometric and quantification analyses of CFSE intensity in CFSE-labeled T cells co-cultured with G-MDSCs from mice treated with ethanol and G-MDSCs, or from mice treated with vehicle (n=6). b), The mRNA level of immune suppressive relating targets IL10, Arginase-1, and GM-CSF determined by q-PCR (n=6). c), The mRNA level of IL-10 in isolated G-MDSCs from mice treated with vehicle or ethanol determined by q-PCR (n=6). Data were analyzed as mean value ± SD and Student's T-test was used to assess the result significance. *p<0.05, **p<0.01, compared with the control group; n.s., not significant.

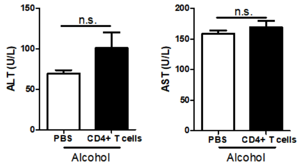

Fig. S3. The ALT and AST level in mice treated with PBS or T cells transfer (n=5). Data were analyzed as mean value ± SD and Student's T-test was used to assess the result significance. n.s., not significant.

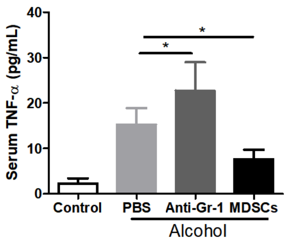

Fig. S4. The serum IL-10 content of mice from control group, ethanol model group, anti-Gr1 treated group and G-MDSCs transferred group (n=6) determined by ELISA kit. Data were analyzed as mean value ± SD and Student's T-test was used to assess the result significance. *p<0. 05, **p<0. 01, compared with ethanol model group, n.s. not significant.

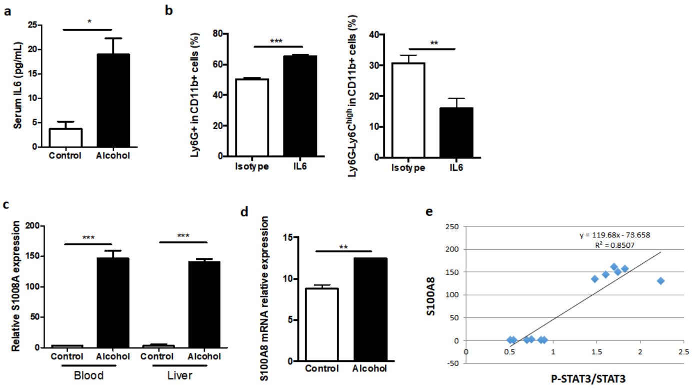

Fig. S5. a), The level of IL-6 in the serum of mice receiving vehicle or ethanol (n=6). b), The population of G-MDSCs and M-MDSCs in bone marrow cells treated with IL6 or isotype antibody (n=6). c), The expression of S100A8 in sorted G-MDSCs from blood and liver of control mice and alcohol-treated mice (n=6). d), The mRNA level of S100A8 in bone marrow cells cultured with vehicle or alcohol (n=6). e), The correlation analysis between the fold changes of pSTAT3/STAT3 and mRNA relative expression of S100A8 in isolated G-MDSCs from the liver of mice (n=6). Data were analyzed as mean value ± SD and Student's T-test was used to assess the result significance. *p<0. 05, **p<0. 01, ***p<0. 001, n.s. not significant.

Supplemental Tables

Supplemental Table 1: The antibody panels used for flow cytometer

Tubes for subpopulation	Co-stained Antibodies
MDSCs	CD11b-APC (clone: M1/70, eBioscience)<br>Ly6G-FITC (clone: 1A8-Ly6g, eBioscience)<br>Ly6C-PE-Cy7 (clone: HK1.4, eBioscience)
T cells for population determination	CD3-APC (clone: 17A2, eBioscience)<br>CD4-FITC (clone: GK1.5, eBioscience)<br>CD8-APC-Cy7 (clone: 53-6.7, BioLegend)
T cells sorted for proliferation assay	CD3-Pacific Blue (clone: 17A2, BioLegend)<br>CD4-PE (clone: RM4-4, BioLegend)<br>CD8-APC-Cy7 (clone: 53-6.7, BioLegend)
Progenitors	CD115-PE<br>CD64-APC<br>CD34-FITC

Supplemental Table 2. Quantitative PCR primers and corresponding sequences.

Name	Forward primer 5' -> 3'	Reverse primer 5' -> 3'
β actin	GGCACCACACCTTCTACAATG	GGGGTGTTGAAGGTCTCAAAC
S100A8	CCGTCTTCAAGACATCGTTTGA	GTAGAGGGCATGGTGATTTCCT
IL10	ACCTGCTCCACTGCCTTGCT	GGTTGCCAAGCCTTATCGGA
CXCL8	ACACTCCACACCTTTCCAT	GGCACACCTCATTTCCATTG
CCL2	GCATCCACGTGTTGGCTCA	CTCCAGCCTACTCATTGGGATCA
CCL4	CCATGAAGCTCTGCGTGTCTG	GGCTTGGAGCAAAGACTGCTG
CCL5	AGATCTCTGCAGCTGCCCTCA	GGAGCACTTGCTGCTGGTGTAG
